# SU5416 induces premature senescence in endothelial progenitor cells from patients with age-related macular degeneration

**Published:** 2011-01-10

**Authors:** Michelle Thill, Marc J. Berna, Frank Kunst, Henning Wege, Natalya V. Strunnikova, Natalya Gordiyenko, Rebecca Grierson, Gisbert Richard, Karl G. Csaky

**Affiliations:** 1 Klinik und Poliklinik für Augenheilkunde, Universitätsklinikum Hamburg-Eppendorf, Hamburg, Germany; 2 Medizinische Klinik I, Universitätsklinikum Hamburg-Eppendorf, Hamburg, Germany; 3 National Eye Institute, National Institutes of Health, Bethesda, MD; 4 Duke University Eye Center, Duke University, Durham, NC

## Abstract

**Purpose:**

We recently demonstrated increased frequency and growth potential of late outgrowth endothelial progenitor cells (OECs) in patients with neovascular age-related macular degeneration (nvAMD). This study investigated the effects of short- and long-term in vitro inhibition of vascular endothelial growth factor (VEGF) Receptor-2 (VEGFR-2) signaling by SU5416 and other inhibitors of the VEGF signaling pathway in OECs.

**Methods:**

OECs, from the peripheral blood of patients with nvAMD, and human umbilical vein endothelial cells were grown in the presence of SU5416, other VEGFR-2 tyrosine kinase inhibitors (TKIs), and inhibitors of phosphatidylinositol 3′-Kinase (PI3K)/protein kinase B (Akt) and protein kinase C (PKC) in complete angiogenic medium. Apotosis was assessed after 48 h using the fluorescein isothiocyanate Annexin V method. Cell counts were performed for 10 days, and features of senescence were analyzed using senescence-associated β-galactosidase staining, the telomeric repeat amplification protocol for telomerase activity, Southern blot analysis for mean telomere length, flow cytometric analysis for cell-cycle arrest, and western blot for p53 and p21. Control OECs, cells treated for 7 days with inhibitors, as well as naturally senescent OECs were analyzed for expression of different endothelial antigens, including VEGFR-2 and the receptor for stromal cell-derived factor 1, chemokine receptor 4 (CXCR-4). Migration in vitro to VEGF and stromal cell-derived factor 1 of OECs was assessed.

**Results:**

SU5416, other VEGFR-2 TKIs, and inhibitors of PI3K, Akt, and PKC induced apoptosis, inhibited long-term proliferation, reduced telomerase activity, and induced premature senescence and cell-cycle arrest in OECs as well as in human umbilical vein endothelial cells. Naturally senescent cells and cells rendered senescent by VEGFR-2 TKIs had reduced VEGFR-2 and CXCR-4 expression and demonstrated reduced migratory ability to VEGF.

**Conclusions:**

This study demonstrates apoptosis upon short-term inhibition and inhibition of long-term survival of OECs from patients with nvAMD by SU5416, presumably via PI3K/Akt and/or PKC-mediated reduction in telomerase activity and subsequent induction of premature senescence, which is accompanied by impaired endothelial activity. Therefore, induction of premature senescence in endothelial cells may represent a potential therapeutic target in nvAMD.

## Introduction

Age-related macular degeneration (AMD) is the leading cause of irreversible visual impairment and blindness in the older population of the developed world [1]. Until recently, it was assumed that cytokines, such as vascular endothelial growth factor (VEGF), promote formation and growth of choroidal neovascularization (CNV), the anatomic correlate of the neovascular form of AMD (nvAMD), by causing pre-existing choroidal endothelial cells to sprout [2]. However, VEGF can also mobilize endothelial progenitor cells (EPCs) from the bone marrow and support differentiation of these EPCs into mature endothelial cells at sites of neovascularization [3-7]. In animal models of nvAMD, several studies now show that a substantial fraction of vascular cells participating in CNV are derived from the bone marrow [8-12]. Clinical evidence for a role of EPCs in the development of CNV comes from the identification of the EPC marker CD133 in specimens of surgically excised CNV [13], detection of an increased number of circulating CD34^+^ hematopoietic cells in patients with nvAMD [14], and our own findings of a significantly increased number of late outgrowth endothelial progenitor cells (OECs) in the peripheral blood of patients with nvAMD [15].

Activation by VEGF of its receptor VEGF receptor-2 (VEGFR-2) promotes proliferation and survival of endothelial cells via the phosphatidylinositol 3′-kinase (PI3K)/protein kinase B (Akt) [16,17] and protein kinase C (PKC) [17,18] signal transduction pathways. Our recent investigations have shown that OECs demonstrate high expression of VEGFR-2 and that their proliferation potential positively correlates with VEGFR-2 expression [15].

Endothelial cells, like most normal somatic cells, manifest a limited proliferation potential [19-21], and when this potential is exhausted, cells enter a physiologic process termed “replicative senescence” (for review see [22]). Mechanistically, repeated cell division is associated with progressive shortening of telomeres, and synthesis of telomeres requires a reverse transcriptase called telomerase. Although somatic cells were thought to rarely possess telomerase activity, endothelial cells stimulated to proliferate in vitro show marked upregulation of telomerase activity [23], regulated by VEGF and other growth factors [23,24], via their intracellular effectors Akt and PI3K [25]. In addition to the alterations in replication, senescent endothelial cells also show other characteristic changes in gene expression, morphology, and function [22,26], for example, a marked reduction in their migratory ability [27-29].

VEGF-neutralizing antibodies are the current treatment standard for nvAMD. Other therapeutical options are being investigated, including selective and nonselective VEGFR-2 tyrosine kinase inhibitors (TKIs) [30-34]. SU5416 was developed as a potent and selective VEGFR-2 TKI [35] and one of the first compounds to be evaluated in large-scale clinical trials [36,37]. It was shown to possess long-lasting inhibitory activity in vitro as well as in vivo [38] and to increase tumor and endothelial cell apoptosis [39] as well as decrease the size of experimental CNV [34]. Therefore, in the present study, SU5416 was chosen to study the in vitro effect of short- and long-term VEGFR-2 inhibition on apoptosis, survival, telomerase activity, and cell-cycle status of OECs from patients with nvAMD. In addition, we investigated the hypothesis that pharmacologically induced premature senescence may result in changes in levels of functional proteins and/or a decrease in endothelial migration, a function vital to the formation of CNV.

## Methods

### Reagents

SU5416, KRN633, KRN951 ZM323881, Wortmannin, Ly 294002, and bisindolylmaleimide I were purchased from Calbiochem (EMD Chemicals, San Diego, CA). Antibodies against p21 and p53 were from Cell Signaling Technology Inc. (Beverly, MA); goat polyclonal antibody to β-actin was used as a loading control (Abcam Inc., Cambridge, MA). Cytokines VEGF and stromal cell-derived factor-1 (SDF-1) were from Peprotech (Rocky Hill, NJ).

### Isolation and culture of late outgrowth endothelial progenitor cells

We have previously shown robust expansion and proliferation of OECs from a subset of patients with nvAMD [15]. These AMD-affected participants were recruited from a population of patients attending the National Eye Institute (NEI) clinic in Bethesda, MD. The protocol for collection and use of human blood samples was approved by the NEI Institutional Review Board, and all participants gave informed consent to participate in the study.

Peripheral blood was collected in a tube system containing sodium heparin and a Ficoll Hypaque solution for separation of blood media (Vacutainer CPT, Becton Dickinson, Franklin Lakes, NJ). After immediate density gradient centrifugation of the preparation, mononuclear cells were resuspended in endothelial growth medium-2 (EGM-2MV; Cambrex, Walkersville, MD), composed of endothelial cell basal medium-2 (EBM-2), 5% fetal bovine serum (FBS), and growth factors (VEGF, basic fibroblast growth factor [b-FGF], insulin growth factor [IGF], and epidermal growth factor [EGF]). Cells were plated at a density of 2×10^6^ cells/cm^2^ in 24-well plates precoated with fibronectin (BD, Bedford, MA). The medium was changed daily for 7 days and on alternate days thereafter according to the protocol established by Lin et al. [29]. OEC clusters, identified as well circumscribed monolayers of cobblestone-appearing cells, began to appear between 7 and 30 days of culture. Subconfluent cells were trypsinized and replated in vessels coated with human fibronectin at a concentration of 10 μg/cm^2^ (Chemicon, Temecula, CA). OECs were further subpassaged and expanded until cell senescence, as determined by morphology changes, decrease in proliferation, and positive staining for senescence-associated β-galactosidase (SA-β-gal) (BioVision Research Products, Mountain View, CA) was reached. Human umbilical vein endothelial cells (HUVEC, Cambrex, Walkersville, MD) were similarly cultured in EGM-2MV medium and on fibronectin-coated vessels. All experiments were performed in EGM-2MV medium to mimic angiogenic conditions and on early passage, actively proliferating, subconfluent nonsenescent cells. Endothelial cell phenotype was confirmed by different methods (immunophenotyping for endothelial cell-surface marker expression, uptake of 1,1'-dioctadecyl-3,3,3′,3′-tetramethylindocarbocyanine perchlorate (DiI)-acetylated low-density lipoprotein, staining for *Ulex europaeus* lectin, and in vitro tube formation assays) as described [15]. Prolonged passaging of OECs and HUVEC was undertaken to obtain cells that had undergone replicative senescence and were used as a control for naturally senescent cells.

To assess cell proliferation under different inhibitory conditions, cells were plated at 10^5^ cells/well in six-well plates. Inhibitor was added every other day, and cells were subcultured to 80% confluency and reseeded at a density of 10^5^ cells/well, with addition of fresh inihibitor. All inhibitors had been dissolved in dimethyl sulfoxide (DMSO). The negative control consisted of DMSO solution (10 μl DMSO/ml EGM-2MV) without inhibitor. Cell counts were performed using a Neubauer counting chamber (Marienfeld, Germany) and trypan blue stain (Invitrogen, Carlsbad, CA) for exclusion of dead cells, according to the manufacturer’s instructions. Cell counts were performed using a Neubauer counting chamber (Marienfeld, Germany). 0.1 ml of trypan blue (Invitrogen, Carlsbad, CA) stock (0.4% solution of trypan blue in buffered isotonic salt solution) was added to 1 ml of cells. The cell suspension was immediately loaded into the counting chamber and cells that had taken up trypan blue were considered non-viable and excluded from counting. All experiments were repeated at least three times.

### Apoptosis assay

Short-term survival of OECs and HUVEC treated with SU5416 and other inhibitory conditions in complete EGM was assessed by collecting floating and adherent cells incubated for 48 h and staining cells with the fluorescein isothiocyanate (FITC) Annexin V/Dead Cell Apoptosis kit according to the manufacturer’s protocol (Invitrogen). In brief, cells treated with different conditions were harvested and washed twice in cold PBS, then resuspended in annexin-binding buffer. FITC annexin V and propidium iodide (PI) were added to the cell supension and cells were incubated at room temperature for 15 min. After the incubation period, annexin-binding buffer, was added an samples were kept on ice until fluorescence activated cell sorting (FACS) measurement. After FACS acquisition, percentage of apoptotic cells (Annexin V positive/Propidium Iodide negative) was assessed using the Flowjo software (Tree Star Inc., Ashland, OR).

### Senescence assay

SA-β-gal activity was detected using the Senescence Detection kit (Biovision, Mountain View, CA). OECs and HUVEC grown on eight-well culture slides and treated with different inhibitory modalities for different time points were fixed and stained according to the manufacturer’s protocol. In brief, cells were fixed for 10–15 min at room temperature, washed twice with PBS, then incubated overnight in staining solution at 37 °C. Fixed cells were observed under a microscope for development of blue color.

### Detection of telomerase activity

Telomerase activity was detected in OECs and HUVEC inhibited with different conditions for 3 or 7 days, using the TeloTAGGG Telomerase PCR ELISA (Roche Applied Science, Indianapolis, IN), which utilizes the telomeric repeat amplification protocol (TRAP). Inhibitor was added every other day, and cells were subcultured to 80% confluency, counted, and re-seeded at a density of 10^5^ cells/well, with addition of fresh inihibitor. The negative control consisted of DMSO solution (10 μl DMSO/ml EGM-2MV) without inhibitor. Reversibility of inhibition of telomerase activity was tested by returning cells previously inhibited for 7 days to complete EGM-2MV medium without inhibitor for another 3 days. Cells were also counted at the time of collection, and telomerase activity was adjusted for cell number.

### Southern blot analysis of mean telomere length

Analysis of mean telomere length of cells inhibited for 7 days was performed as previously published [40]. Briefly, genomic DNA was isolated from harvested cells, electrophoresed, blotted and transferred to positively charged Magnacharge membranes (Osmonic, Minnetonk, MA). Membranes were hybridized with ^32^P-(TTAGGG)3 as a telomeric probe using Hybrisol II (Intergen, Purchase, NY). Mean terminal restriction fragment (TRF) length was determined from. TRF length was determined from scanned autoradiographs by integrating the signal intensity above background over the entire TRF distribution, using ImageQuaNT software (Molecular Dynamics, Sunnyvale, CA).

### Western blotting

For western blot analysis for p21 and p53, cells subjected to inhibitory treatment for 7 days were lysed in lysis buffer containing 50 mM Tris/HCl (pH 7.5), 150 mM NaCl, 1% Triton X-100, 1% deoxycholate, 0.1% sodium azide, 1 mM ethylene glycol tetraacetic acid (EGTA), 0.4 mM EDTA, 0.2 mM sodium orthovanadate, 1 mM phenylmethylsulfonyl fluoride (PMSF), and one protease inhibitor tablet (Roche, Basel, Switzerland) per 10 ml. After sonication, lysates were centrifuged at 10,000× g at 4 °C for 15 min, and protein concentration was measured using the Bio-Rad protein assay reagent (Bio-Rad, Hercules, CA). Equal amounts of lysates were subjected to sodium dodecyl sulfate PAGE using 10% Tris-glycine gels (Invitrogen). After electrophoresis, protein was transferred to nitrocellulose membranes. Membranes were blocked in blocking buffer (50 mM Tris/HCl (pH 8.0), 2 mM CaCl_2_, 0.05% Tween-20, and 5% nonfat dry milk) at room temperature for 1 h. Membranes were then incubated with 1:1,000 diluted rabbit monoclonal Antibody (mAb) against p21, 1:1,000 diluted antimouse p53, or 1:500 goat polyclonal antibody to β-actin-loading control at 4 °C overnight and under constant agitation. After primary antibody incubation, membranes were washed twice in blocking buffer for 4 min, followed by incubation with horseradish peroxidase (HRP)-conjugated antirabbit or antigoat secondary antibody Santa Cruz (Santa Cruz, CA) at room temperature for 45 min and under constant agitation. Membranes were washed twice in blocking buffer for 4 min, twice in washing buffer (50 mM Tris/HCl (pH 8.0), 2 mM CaCl_2_, 80 mM NaCl, 0.05% Tween-20) for 4 min, incubated for 4 min with chemiluminescence detection reagents (Pierce, Rockford, IL), and finally exposed to Kodak Biomax film (Kodak, Rochester, NY).

### Flow cytometric analysis for cell cycle status (FACS) analysis for cell-cycle status

OECs and HUVEC subjected to inhibitory conditions for 7 days were collected (floating and adherent cells combined) and stained with Vybrant DyeCycle Green (Molecular Probes, Eugene, OR) according to the manufacturer’s protocol. Briefly, 2 μl of Vybrant DyeCycle Green stain was added to flow cytometry tubes each containing 1 ml of cell suspension, followed by incubation at 37 °C for 30 min, protected from light. Stained samples were immediately analyzed by FACS using 488 nm excitation and green emission. After FACS acquisition, cell-cycle analysis was performed using the cell-cycle platform of the Flowjo software.

### Flow cytometric analysis for endothelial cell markers analysis for endothelial cell markers

For FACS analysis of nonsenescent OECs, naturally senescent OECs, and cells rendered prematurely senescent for 7 days by inhibitory methods, mAbs against CD31-FITC, CD146-phycoerythrin (PE), Inter-Cellular Adhesion Molecule (ICAM)-1 and −2-PE and CXCR-4-PE (all BD PharMingen, San Jose, CA) and VEGFR-2/Kinase insert domain receptor (KDR)-PE (R&D Systems Inc., Minneapolis, MN) were used. Isotype-matched immunoglobulin G (IgG) antibodies were used as a control. OECs and HUVEC were trypsinized and incubated at 4 °C for 30 min with primary or isotype control antibody, washed, and acquired by FACS. Analysis was performed using the FlowJo software.

### Migration assays

The migratory ability of nonsenescent OECs, naturally senescent OECs, and cells rendered prematurely senescent after 7 days of treatment with VEGFR-2 inhibitors was assessed using a commercially available modified Boyden chamber assay (BD BioCoat Angiogenesis System: Endothelial Cell Migration, Becton Dickinson, Franklin Lakes, NJ). After serum starvation overnight in EBM-2 + 0.1% BSA, cells suspended in EBM-2+0.4% FBS were placed in the upper chamber, while the lower chamber contained either 5 ng/ml VEGF in EBM-2+0.4% FBS, 500 ng/ml SDF-1 in EBM-2+0.4% FBS, or complete EGM-2MV. Cells were labeled using the Calcein acetoxymethyl ester (AM) dye (Invitrogen) after 22 h of migration, and a fluorescence plate reader (Synergy microplate reader, Biotek, Winooski, VT) was used to quantify the migrated cells.

### Statistical analysis

All experiments were performed at least three times. Data are presented as mean±standard error of the mean (SEM) and were analyzed with the Student *t* test for paired data using the software StatView (SAS Institute, Casy, NC). P values <0.05 were considered significant.

## Results

### Induction of apoptosis upon short-term treatment with SU5416

**Figure 1:**
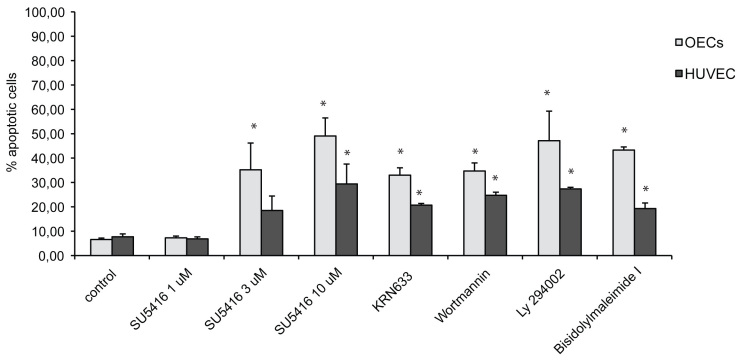
Effect of 48 h of inhibition of outgrowth endothelial progenitor cells and human umbilical vein endothelial cells on percentage of apoptotic cells, as determined by flow cytometric analysis of Annexin V fluorescein isothiocyanate positive/propidium iodide (PI) negative cells. Cells were grown in complete angiogenic medium (EGM-2MV) control conditions (10 μl DMSO/ml EGM-2MV), or in the presence of 1, 3, and 10 μM SU5416, 4 μM KRN633, and inhibitors of the downstream mediators Akt (100 nM Wortmannin), PI 3-kinase (5 μM Ly 294002), and PKC (1 μM bisindolylmaleimide I). The graphs represent the mean±SEM cell number from three independent (OECs were derived from three different patients, for HUVEC analysis was repeated at least twice) experiments. Student’s *t*-test for paired data was used for statistical comparison between control and inhibitory conditions. P values <0.05 were considered significant and are denoted by an asterisk in the figure.

As shown in Figure 1, untreated HUVEC and OEC cultures contained relatively low levels (7.7±2.6% and 6.6±1.5%, respectively) of apoptotic (Annexin V-positive, PI-negative) cells. When increasing concentrations of SU5416 as well as another VEGFR-2 TKI (4 μM KRN633) and inhibitors of the Akt (Wortmannin), PI3K (Ly 294002), and PKC (bisidolylmaleimide I) pathways were added for 48 h, the percentage of Annexin V-positive cells was significantly increased compared to control cells, especially in OECs.

### Decrease in proliferation upon long-term treatment with SU5416

**Figure 2:**
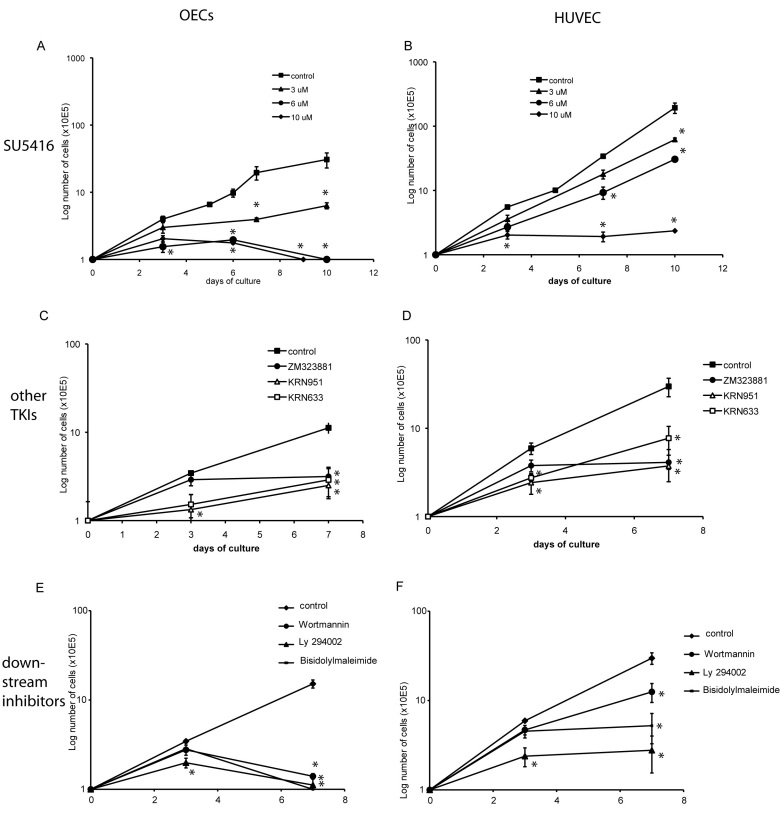
Effect of different inhibitory conditions on long-term survival of late outgrowth endothelial progenitor cells and human umbilical vein endothelial cells (HUVECs). Effect of SU5416 on proliferation of OECs (**A**) and HUVEC (**B**) in complete angiogenic medium (EGM-2MV). Cells were grown in EGM-2MV control conditions (10 μl dimethyl sulfoxide [DMSO]/ml EGM-2MV), or in the presence of 3, 6, and 10 μM SU5416 added every 48 h. Long-term survival of OECs (**C** and **E**) and HUVEC (**D** and **F**) was similarly assessed in the presence of other VEGFR-2 Tyrosine kinase inihbitors (TKIs; 10 μM ZM323881, 3 μM KRN951, 4 μM KRN633) and in the presence of inhibitors of the downstream mediators Akt (100 nM Wortmannin), PI 3-kinase (5 μM Ly 294002), and PKC (1 μM bisindolylmaleimide I). All cell counts were performed using the trypan blue exclusion method and a Neubauer counting chamber. The graphs represent the mean±SEM cell number for three independent experiments (OECs derived from three independent (OECs were derived from three different patients, for HUVEC analysis was repeated at least twice) experiments. Student’s *t*-test for paired data was used for statistical comparison between control and inhibitory conditions at each time point. P values <0.05 were considered significant and are denoted by an asterisk in the figure).

To analyze the fate of OECs and HUVEC upon long-term inhibition of VEGFR-2 and its downstream signaling pathways, inhibitors were added to the medium every other day for up to 10 days. Treatment with SU5416 resulted in a dose-dependent decrease in proliferation of OECs (Figure 2A). Generally, HUVEC demonstrated a higher proliferation rate when compared to OECs, and proliferation of HUVEC was only decreased or inhibited when higher concentrations of SU5416 were used (Figure 2B). Other TKIs of VEGFR-2 demonstrated similar inhibition of OEC and HUVEC long-term proliferation (Figure 2C,D). Inhibitors of VEGF/VEGFR-2 downstream mediators, such as Akt (Wortmannin), PI3K (Ly 294002), and PKC (bisindolylmaleimide I) also markedly inhibited OEC and HUVEC proliferation in complete angiogenic medium (Figure 2E,F).

### Induction of premature senescence by SU5416 and other inhibitors

**Figure 3:**
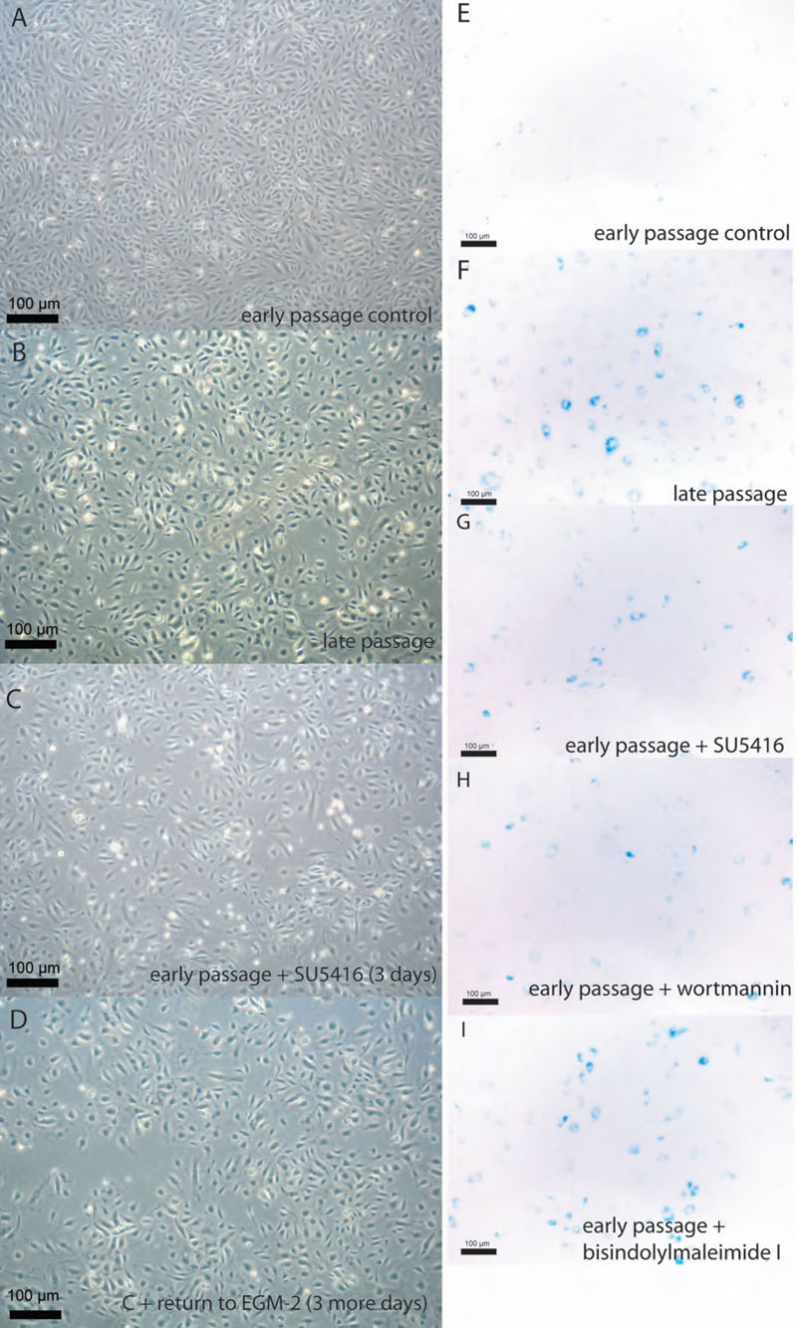
Representative images of morphology of early passage control late outgrowth endothelial progenitor cells in complete angiogenic medium containing dimethyl sulfoxide (10 μl/ml; **A**), late passage OECs (**B**), control early passage OECs after 3 days of inhibition with 10 μM SU5416 (**C**), and 3 days after returning SU5416 treated cells to EGM-2MV without inhibitor (**D**) are shown. Representative images of senescence-associated beta-galactosidase staining (blue color) in early passage control OECs (**E**), late passage OECs (**F**) and early-passage OECs treated with either 10 uM SU5416 (**G**), 100 nM Wortmannin (**H**) or 1 uM Bisindolylmaleimide I (**I**) for 3 days are shown.

After ex vivo expansion, OECs from all patients as well as HUVEC eventually (late passages) became senescent, as demonstrated by a decrease in proliferation rate, morphological changes (Figure 3B), and positive staining for SA-β-gal (Figure 3E).

Early passage OECs and HUVEC were grown under inhibitory conditions as previously described, and experiments were terminated after either 3 or 7 days for cytochemical analysis of SA-β-gal expression. SA-β-gal expression is a common feature of senescent cells [41], including senescent endothelial cells [42]. Morphological signs of senescence, such as decreased cell density and enlarged and flattened cell morphology, as well as increased SA-β-gal expression appeared in single OECs after 3 days of inhibitory conditions and became manifest in the majority of cells after 6 to 7 days of inhibition. Inhibition for 3 days with SU5416 (Figure 3C,G) and the inhibitors of Akt (Figure 3H), PI3K (not shown), and PKC (Figure 3I) pathways induced senescent morphology and expression of SA-β-gal in OECs. To demonstrate irreversibility, cultures inhibited for 7 days were returned to EGM-2MV medium without inhibition and cultured for at least 3 more days. Cells previously treated with inhibitors maintained proliferation arrest and retained senescent morphology (Figure 3C) and SA-β-gal expression upon replacement of growth conditions with fresh EGM-2MV medium (not shown). Similar results were obtained with HUVEC (data not shown).

### Decrease of telomerase activity after treatment with SU5416

**Figure 4:**
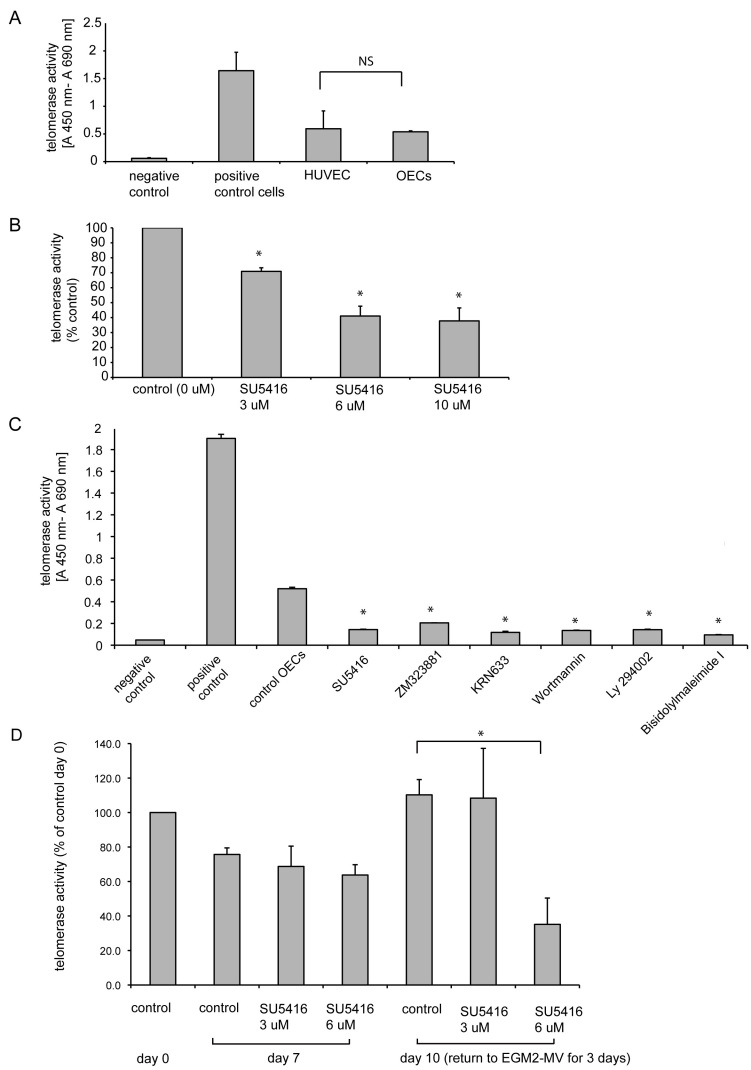
Analysis of telomerase activity as assayed by the telomeric repeat amplification protocol. **A**: Telomerase activity was detected in early passage nonsenescent late outgrowth endothelial progenitor cells (OECs) and Human Umbilical Vein Endothelial Cells (HUVEC) cultured in complete angiogenic medium (EGM-2MV). Negative control represents telomerase activity of heat-inactivated OEC samples, positive control corresponds to the tumor cell sample provided by the manufacturer (TeloTAGGG Telomerase PCR ELISA, Roche Applied Science, Indianapolis, IN). NS denotes not statistically significant difference in telomerase activity between HUVEC and OECs. **B**: Telomerase activity is decreased in OECs treated with 3, 6 and 10 μM SU5416 for 3 days compared to control EGM-2MV (with the addition of 10 μl DMSO/ml, 0 μM SU5416). **C**: Telomerase activity is decreased in OECs treated with EGM-2MV supplemented with inhibitors SU5416 (6 μM), ZM323881 (10 μM), KRN633 (4 μM), Wortmannin (100 nM), Ly 294002 (5 μM) and Bisindolylmaleimide I (1 μM) for 3 days compraed to control medium (EGM-2MV with the addition of 10 μl DMSO/ml). **D**: Recovery of telomerase activity is dose-dependent. Telomerase activity was assessed for control OECs at day 0 and 7, OECs inhibited for 7 days with SU5416 3 μM and 6 μM and for OECs returned to EGM-2MV without inhibitor for another 3 days after 7 days of inhibition. Graphs **A** and **C** represent the mean±SEM telomerase activity, **B** and **D** the mean percentage in telomerase activity as compared to control (medium without inhibitor in **B**, medium without inhibitor after 10 days of culture in **D**), for three independent experiments each (OECs derived from three different patients). Student’s *t*-test for paired data was used for statistical comparison between control and inhibitory conditions. * indicates a statistically significant difference (p<0.05) as compared to control.

We then tested whether these functional and morphological signs of senescence were preceded by changes in telomerase activity. First, telomerase activity in nonsenescent early-passage OECs and HUVEC cultured in EGM-2MV medium was assessed using TRAP. Telomerase activity was present in OECs and HUVEC to a similar extent (Figure 4A). Telomerase activity was then analyzed after 3 or 7 days of inhibitory treatments. Treatment with SU5416 for 3 days suppressed telomerase activity in OECs in a dose-dependent manner (Figure 4B). Telomerase activity was also decreased after inhibition of OECs with other VEGFR-2 TKIs and inhibitors of VEGF downstream signals Akt (Wortmannin), PI 3-kinase (Ly 294002), and PKC (bisindolylmaleimide I; Figure 4C). Telomerase activity was similarly decreased in HUVEC (data not shown) and remained decreased in both OECs and HUVEC after 7 days of inhibition (see Figure 4D for representative experiment). After returning inhibited cells to complete medium without inhibitor at day 7, telomerase activity demonstrated a concentration-dependent recovery at day 10 with reduction of telomerase activity being irreversible at higher concentrations (full recovery at 3 μM, no recovery at 6 μM).

### Lack of shortening of telomere length after SU5416 inhibition for 7 days

**Figure 5:**
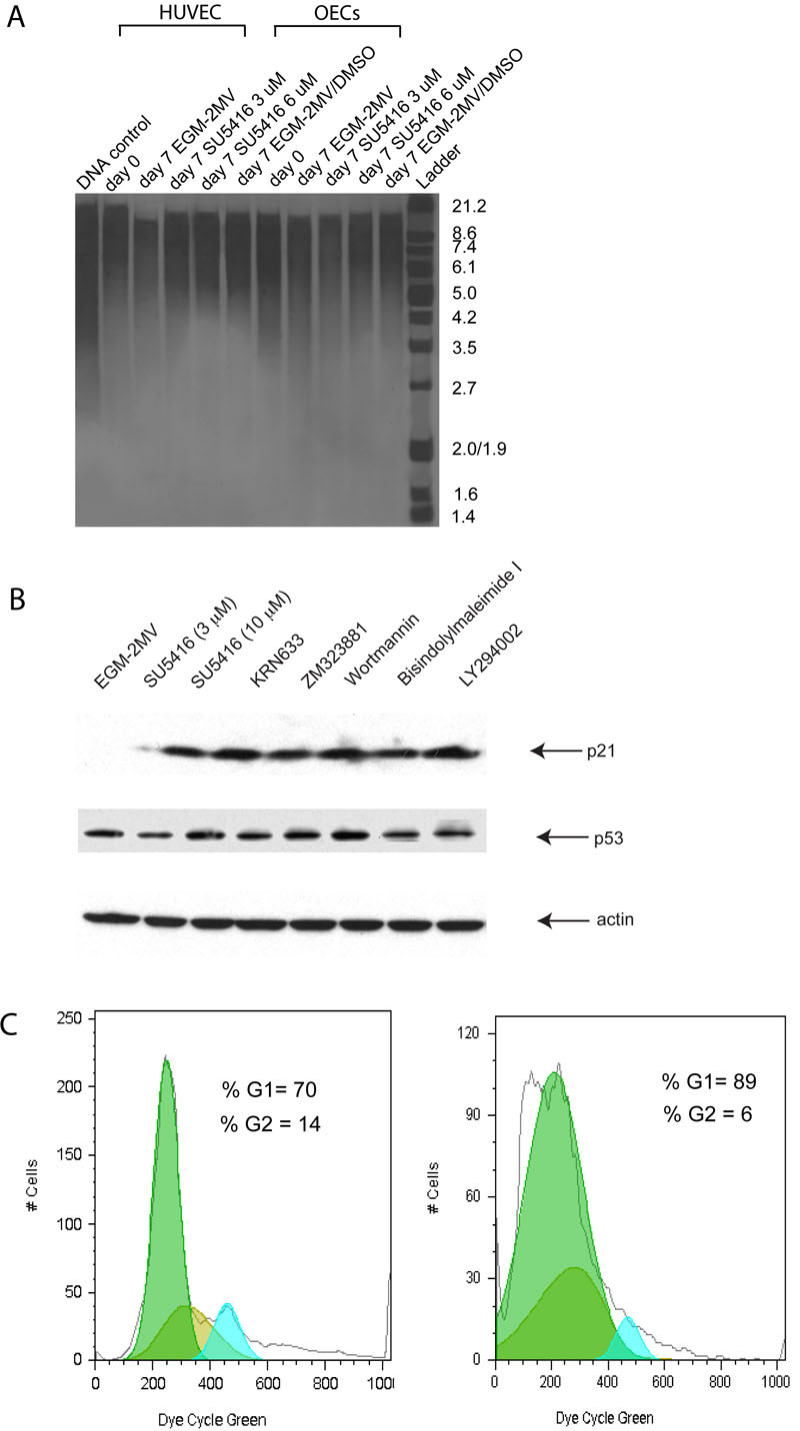
Analysis of telomere length, cell cycle proteins p21 and p53 and cell cycle status. **A**: A representative Southern blot analysis of telomere length of control Human Umbilical Vein Endothelial Cells (HUVEC) and late outgrowth endothelial cells (OECs) (in EGM-2MV or EGM-2MV with the addition of 10 μl DMSO/ml) and HUVEC and OECs inhibited with SU5416 at 3 and 6 μM for 7 days is shown. **B**: A representative western blot analysis is given for p21 and p53 in OECs treated with different inhibitory conditions: control=EGM-2MV with the addition of 10 μl DMSO/ml, EGM-2MV supplemented with inhibitors SU5416 at 3 and 10 μM, KRN633 (4 μM), ZM323881 (10 μM), Wortmannin (100 nM), Bisindolylmaleimide I (1 μM) and Ly 294002 (5 μM). Upper lane represents p21, second lane represents p53 and lower lane represent corresponding beta-actin loading controls. **C**: Presents a representative flow cytometric cell cycle analysis of OECs treated with SU5416, using Vybrant DyeCycle green. Left graph represents control OECs (EGM-2MV with the addition of 10 ul DMSO/ml), right graph represents OECs treated with SU5416 (6 μM) for 7 days. Green=G0/G1 peak area, blue=G2 peak area.

Southern blot analysis did not reveal shortening of telomere length after 7 days of inhibition with SU5416 in HUVEC or OECs as compared to day 0 or day 7 controls (representative Southern blot and TRF length in Figure 5A).

### Upregulation of p21 and cell cycle arrest after treatment with SU5416

Western blot analysis for p21 in OECs treated for 7 days revealed marked upregulation of p21 in response to SU5416 as well as other VEGFR-2 inhibitors and Akt, PI-3-K, and PKC inhibition (Figure 5B). p53 remained unchanged in all conditions.

To study the cell-cycle status of cells treated with SU5416, cells were incubated with the DNA-selective Vybrant DyeCycle Green stain and frequency histograms were generated to reveal the phases of the cell cycle. SU5416 caused profound changes in the cell-cycle status after 7 days of treatment, as revealed by an arrest of cells in the cell cycle phase G0/G1 (Figure 5C).

### Decrease of endothelial antigen expression and migratory ability

**Figure 6:**
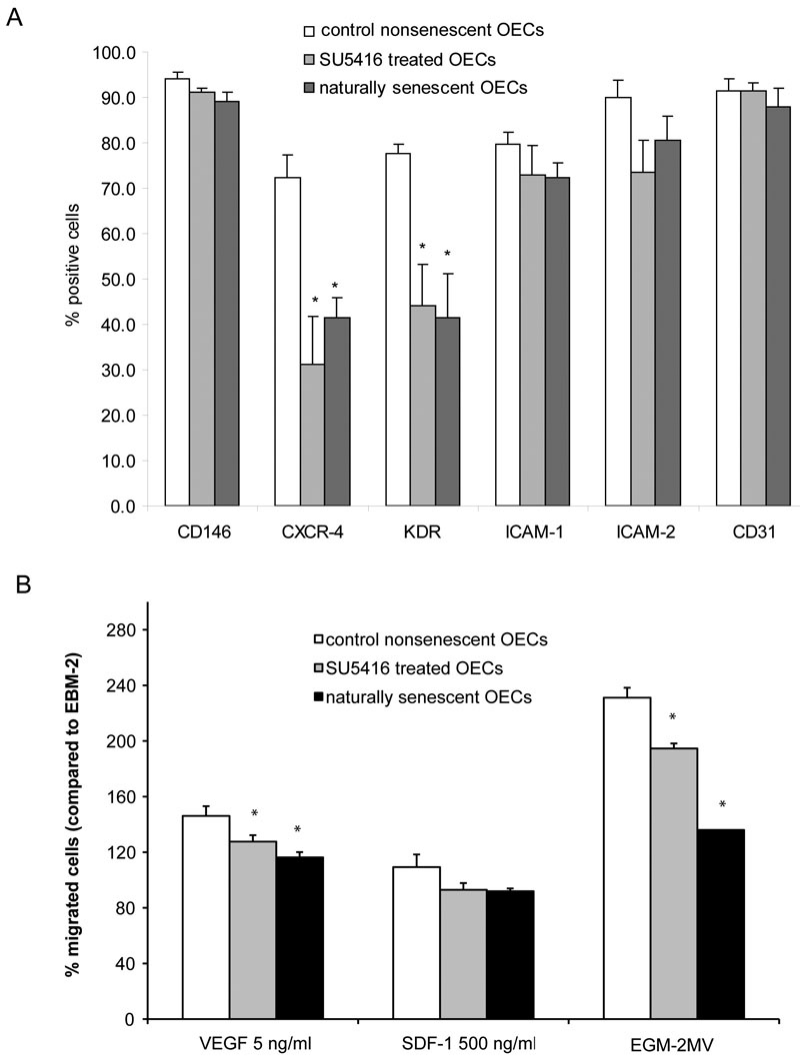
Effect of senescence on antigen expression and migration of late outgrowth endothelial cells (OECs) and Human Umbilical Vein Endothelial Cells (HUVEC). **A**: Expression of different antigens by control nonsenescent OECs, OECs treated with SU5416 (6 μM) for 7 days and late-passage naturally senescent OECs was detected by flow cytometry. The graphs represent the mean±Standard error of the mean (SEM) percentage of positive cells for OECs derived from three different patients. * indicates a statistically significant difference (p<0.05) as compared to control OECs. **B**: Migration of control OECs, OECs treated with SU5416 (6 μM) for 7 days and late-passage naturally senescent OECs to Vascular Endothelial Growth Factor (VEGF), Stromal Cell derived Factor-1 (SDF-1) and complete angiogenic medium (EGM-2MV) is shown. The graphs represent the mean±SEM percentage of migrated cells (as compared to cells migrated to EBM-2 basal medium without the addition of serum or growth factors) for OECs derived from three different patients. * indicates a statistically significant difference (p<0.05) as compared to control OECs.

Flow cytometric analysis was performed to detect differences in endothelial cell protein expression in cells that had become naturally senescent after repeated passaging or prematurely senescent (as determined by morphological aspect, proliferation arrest, and SA-β-gal expression) during VEGFR-2 inhibition. Melanoma cell adhesion molecule/CD146, Platelet Endothelial Cell Adhesion Molecule-1/CD31, ICAM-1, and ICAM-2 are adhesion proteins participating in the recruitment of leukocytes to sites of tissue injury and inflammation. VEGFR-2 and CXCR-4, the receptor for SDF-1, are both implicated in the migration of endothelial cells and the recruitment of progenitor cells into neovascular tissues [7,43-45]. Analysis revealed no statistically significant difference in levels of CD146, CD31, ICAM-1, and ICAM-2 between nonsenescent, naturally senescent, and prematurely senescent OECs. VEGFR-2 and CXCR-4 expression levels, however, were significantly reduced in naturally senescent OECs and OECs rendered prematurely senescent by treatment with SU5416 for 3 days compared to nonsenescent OECs (Figure 6A). The same observation was made for HUVEC and other VEGFR-2 inhibitors (data not shown).

VEGFR-2 and CXCR-4 are involved in endothelial cell migration via their ligands VEGF and SDF-1. We therefore performed an in vitro migration assay toward VEGF and SDF-1 to analyze for differences in migratory ability between nonsenescent, naturally senescent, and prematurely senescent cells (after inhibitory treatments). The migration toward VEGF and EGM-2MV medium of naturally senescent OECs and OECs rendered prematurely senescent by SU5416 treatment was significantly reduced compared to nonsenescent OECs (Figure 6B). While there was a trend toward reduced migration to SDF-1 attractant, a statistically significant difference between treatment groups could not be revealed. Migration assays involving HUVEC gave similar results (data not shown).

## Discussion

The results of this study indicate that blocking of the VEGF receptor-2 signaling with the potent, selective, and long-lasting compound SU5416 inhibits survival of OECs isolated from patients with nvAMD as well as HUVEC by inducing apoptosis upon short-term exposure and premature senescence and cell-cycle arrest upon long-term exposure. The mechanism by which SU5416 as well as other VEGFR-2 TKIs accelerate OEC senescence appears to occur through telomerase inactivation as early as 3 days after initiation of inhibition. Possibly, telomerase inactivation is mediated through the PI3K/Akt and PKC pathways, as inhibition of PI3K/Akt or PKC similarly results in senescence in these cells. Replicative senescence or premature senescence induced by inhibitors is accompanied by impairment of OEC activity, as evidenced by a significantly reduced migratory ability.

Apoptosis and premature senescence seem to be two parallel outcomes activated after cells suffer irreparable damage. How the cells choose between these two responses may be dependent on the cell type, cell-cycle phase [46], the degree of stress [47], or the age of cells [48].

Accelerated or premature senescence is increasingly found to be a response of tumor cells to several chemotherapeutic agents and radiation (for review see [49]). Inhibition of telomerase activity, which is activated in tumor cells, seems to be an attractive target in cancer therapy [50,51]. Once thought to be cancer-cell specific, telomerase activity was found to be upregulated in endothelial cells too, leading to a delay in replicative senescence of these cells [23,24,52]. Moreover, VEGF-dependent activation of telomerase was also observed in vivo where it was required for development of new capillaries in ischemic tissue [53]. Therefore, induction of premature endothelial cell senescence might be an interesting target in anti-angiogenic therapy, e.g., for nvAMD.

Several previous studies have demonstrated acceleration of senescence and proliferation arrest of EPCs and mature endothelial cells in response to different stimuli [25,54-56]. Mechanisms that were identified in replicative as well as in prematurely induced senescence included inactivation of telomerase activity [25,54,56], inhibition of PI3K/Akt [25], modulation of cell-cycle regulatory proteins [56,57], and cell-cycle arrest [57]. We herein demonstrate that induction of premature senescence of OECs by SU5416 involves reduction of telomerase activity, increased expression of p21, and G1 cell-cycle arrest. After 7 days of inhibition, shortening of telomeres was not yet observed in this study. We also demonstrate that direct inhibition of PI3K/Akt and PKC, which are downstream signal transducers of VEGF and mediate proliferation and survival signals in endothelial cells [16,58], similarly induce premature senescence, reduction of telomerase activity, and increased expression of p21. These results suggest that induction of premature senescence by SU5416 and the other TKIs that were used in this study may be through inhibition of these intracellular mediators.

It remains to be determined whether premature senescence is mediated by selective inhibition of VEGFR-2 phosphorylation. SU5416, although considered to be a selective TKI, also exhibits concentration-dependent inhibition of other growth factor receptors, such as the fibroblast growth factor (FGF) receptor, VEGF receptor 1 (flt-1) [59], insulin-like growth factor I receptor, Stem Cell Factor Receptor c-kit [60], and hepatocyte growth factor receptor as well as intracellular kinases, such as sarcoma (Src) [35,61]. Thus, SU5416 and the other TKIs may well induce premature senescence by acting on several growth factor-mediated pathways or even by other unknown mechanisms independent of the tyrosine kinases.

Following irreversible growth arrest, little is known about the fate of senescent endothelial cells. First, it is not clear how premature senescence and apoptosis relate to each other. In one report, senescent HUVEC, arrested in the G1 phase of the cell cycle, displayed a considerable increase in spontaneous apoptosis [57] and were also more prone to drug-induced apoptosis [62], suggesting that senescence may facilitate apoptosis. In another report, the baseline rate of apoptosis remained unchanged during the process of senescence [63]. Second, do senescent cells remain metabolically active and do they retain functional properties? Senescent fibroblasts mixed with transformed epithelial cells stimulated the growth of the latter in vitro and in tumor models [64]. Tumor cells senescing in response to chemotherapy secreted proteins with anti-apoptotic, mitogenic, and angiogenic activities [65]. On the positive side, senescent cells may also inhibit growth of tumor or other neighboring nonsenescent cells by secreting growth inhibitory substances [65,66]. Microarray analysis demonstrated overexpression of inflammatory and immune-response genes in early-passage HUVEC, while those genes were repressed at senescence [67]. We have shown that senescent OECs have decreased levels of VEGFR-2 and CXCR-4, which could result in a lesser responsiveness to the ligands, as demonstrated by reduced migratory ability to EGM-2MV and to VEGF alone. In senescent OECs, we did not find changes in endothelial adhesion molecules, such as ICAM-1, a key protein in leukocyte transendothelial migration previously reported to accumulate in senescent endothelial cells [68].

Finally, whether senescence is a feature of endothelial cells in advanced CNV and whether treatment directed against nvAMD may induce premature senescence of the endothelial subtypes within active CNV has not been studied to date. Therefore, further studies will be directed at analyzing features of senescence in endothelial cells inside experimental CNV as well as a possible induction of premature senescence in vivo by different treatment strategies, especially those directed against the VEGF/VEGFR-2 signaling pathway.

Presumably, induction of premature senescence in endothelial cells involved in the development of CNV may be an important therapeutic target and/or a determinant of treatment response in nvAMD.
